# MicroRNA-214-3p inhibits the stem-like properties of lung squamous cell cancer by targeting YAP1

**DOI:** 10.1186/s12935-020-01506-2

**Published:** 2020-08-27

**Authors:** Tingting Lu, Ying Yang, Ziming Li, Shun Lu

**Affiliations:** Department of Shanghai Lung Cancer Center, Shanghai Chest Hospital, Shanghai Jiao Tong University, 241 Huaihai West Road, Shanghai, 200030 People’s Republic of China

**Keywords:** MiR-214-3p, YAP1, CSC properties

## Abstract

**Background:**

Emerging evidence reveals that microRNAs (miRNAs) play a crucial role in tumor progression, but the underlying mechanism of microRNAs in lung squamous cell cancer (LSCC) remains unclear.

**Method:**

Western-blotting and quantitative real-time PCR (q-PCR) were carried out to detect mRNA and protein expression. Cell proliferation was evaluated by Cell Counting Kit-8 (CCK-8), colony-forming assay or sphere-forming assay, respectively.

**Results:**

MiR-214-3p was markedly de-regulated in LSCC tissues and was inversely related to the level of Yes-associated protein1 (YAP1), which is the core transcription regulator of the Hippo signaling pathway. Kaplan–Meier survival curves illustrated that patients with high miR-214-3p expression demonstrated more favorable clinical outcomes. MiR-214-3p overexpression (OE) repressed proliferation and cancer stem-like cells (CSCs) properties in vitro and in vivo xenograft mouse model. Mechanistically, luciferase activity assay revealed that miR-214-3p directly targets YAP1 by specifically binding on the 3′ UTR of YAP1.

**Conclusion:**

MiR-214-3p plays a pivotal role in CSCs properties by targeting YAP1, which provides a potential treatment strategy for LSCC patients.

## Background

Globally, lung cancer (LC) has high mortality rates [1]. Targeted drugs have been developed in lung adenocarcinoma (AC) to inhibit tumor progression, which remarkably changed treatment strategies and brought positive news to patients with EGFR mutation and ALK or ROS1 rearrangements. Compared with AC, the mainstream treatment regimens for lung squamous cell cancer (LSCC) are still platinum-containing chemotherapeutics and immune therapy, and the targeted therapy for advanced LSCC is rare. Therefore, it is urgent to clarify the underlying mechanism of LSCC to develop new and innovative treatment strategies for LSCC patients.

MicroRNAs are a cluster of noncoding RNAs that modulate gene expression [2]. Recently, microRNAs became known to regulate tumorigenesis. Increasing evidence has shown that aberrant miRNA expression participates in diverse pathogenic processes, such as tumor apoptosis and CSC properties [3–6]. Moreover, microRNA-controlled cancer stem cells (CSCs) have caused widespread concern as various studies link miRNAs to the characteristics of CSCs and differentiation of embryonic stem cells [7–9]. For instance, miR-205 OE remarkably decreases the proliferation of CSCs in human pancreatic cancer [10] while low expression of miR-613 contributed to the expansion of liver CSCs by enhancing the dedifferentiation of hepatoma cells [11].

Hippo is a traditional signaling pathway that regulates various biological processes in both drosophila and human species [12, 13]. The Hippo pathway components involve a major kinase cascade and scaffold proteins and YAP1 is the core transcription regulator of the Hippo signaling pathway. Briefly, in Hippo pathway, Mammalian Sterile20-like Kinase1/2 (Mst1/2) forms a complex with the adaptor protein Salvador1 (Sav1) and ultimately results in YAP1 phosphorylation. Furthermore, phosphorylated YAP1 is sequestered in the cytoplasm and then degraded through the ubiquitin–proteasome [14]. Increasing evidence suggests that high levels of the YAP1 expression are present in distinct human tumors such as in colon, liver, ovarian, cholangiocyte, and prostate cancers [15–18]. Our group previously demonstrated the contribution of YAP1 to the maintenance of CSC activities in FGFR1-amplified lung squamous cell cancer [19].

In this study, to elucidate the function of miRNA in LSCC initiation and progression, we detected the selected miRNA expression profile in 20 LSCC human samples using the q-PCR (Additional file 1: Figure S1). Our results highlighted the low expression of miR-214-3p in human LSCC samples compared to adjacent normal samples. For the first time, we reported that miR-214-3p inhibited CSC capacity by targeting YAP1 in LSCC. Furthermore, we verified that miR-214-3p took part in activating the Hippo signaling activity. Altogether, we confirmed that miR-214-3p exhibited as a tumor suppressor in LSCC initiation, and it also serves as a possible target for LSCC.

## Materials and methods

### Cell lines

The human H520 and HCC95 cell lines were purchased from the American Type Culture Collection. MiR-214-3p and Negative Control (NC) inhibitors and mimics were from Ribobio Technology.

### Human tissue specimens

For research use, the LSCC samples were collected from the Department of Oncology, Shanghai Chest Hospital. Written consent forms were obtained from every LSCC patient. The Institute Research Ethics Committee of Shanghai Chest Hospital approved all protocols.

### Western blotting

YAP1(#4912), LATS1(#9153), p-YAP1(#4911), Vimentin(#12020), Snail(#3879),and Tubulin(#2148) antibodies were all from Cell Signaling Technology (CST, Danvers, MA). OCT4 (ab19875), CD133 (ab19898) and SOX2 (ab171380) were from Abcam. Protein was obtained from cells by using Radio-Immunoprecipitation Assay (RIPA) (Sigma-Aldrich). Equal quantities of protein lysates were loaded on the Sodium dodecyl sulfate–Polyacrylamide Gel Electrophoresis gels (SDS-PAGE gels), and gel electrophoresis was applied to transfer the protein onto a nitrocellulose membrane. The protein strips were ultimately detected by enhanced chemiluminescence (Thermo Fisher Scientific) and captured using the Odyssey Infrared Imaging Analysis Software.

### qRT-PCR

Through the Trizol Reagent (Life Technologies, CA, USA), the total RNA was collected. According to the manufacturer’s protocol, the q-PCR was conducted using the SYBR (Synergy Brands Synergy Brands) Premix Ex Taq Kit (TaKaRa). Briefly, the cDNA (complementary DNA) was used as the template for qPCR detection using TaqMan Technology on an Applied Biosystems 7000 sequence detection system (Applied Biosystems, Foster City, CA). Then we detected the expression of YAP1, SOX2 (SRY-related HMG-box 2), CD133, OCT4 (Octamer-binding transcription factor-4) and NANOG genes by using commercially available primer and probe sequences (Applied Biosystems).

### miRNA transfection

The miRNA mimics were synthesized through the Ribobio Technology. Both the H520 and HCC95 cells were grown in 6-well plates and transfected with Lipofectamine 3000 (Invitrogen; Thermo Fisher Scientific) in Opti-MEM (Invitrogen; Thermo Fisher Scientific). After 48 h, cells were harvested to the following experiments.

### Luciferase activity assay

The luciferase assay was performed using the dual-Luciferase^®^ reporter assay kit (Promega, Madison, WI). Briefly, the 293T cells were grown in 24-well plates and co-transfected with miR-NC or miR-214-3p mimics, pmirglo-H-YAP1-3′ Untranslated Region (UTR) wide type (WT) or pmirglo-H-YAP1-3′UTR-mutation type (Mut) or pGL3-YAP1 promoter, and renilla luciferase vector with Lipofectamine 2000 (Invitrogen). After 36 h, the firefly luciferase activity normalized to the renilla luciferase.

### CCK8 assay

Using the Cell Counting Kit-8 (CCK8) (Dojindo, Kumamoto, Japan), cell proliferation and viability were measured. After transfection, cells were grown into 96-well plates (1000 cells/well, N = 4). The optical density value (OD value) at 450 nm of the cells at the specified time was recorded.

### Colony-forming assay

All 500 cells with different treatments were grown into 6-well plates. The cells were cultured for at least 10 days until the colonies became visible and noticeable. The cells were washed with phosphate buffer saline (PBS), fixed with 4% polyformaldehyde (Sigma-Aldrich, St. Louis, MO), and then stained with Giemsa (Solarbio, Beijing, China). Image J recorded photos of the colonies.

### Sphere formation assay

Briefly, adherent H520 and HCC95 cells with different treatments were seeded into the 6-well ultra-low attachment plates at a density of 3000/well. After 14 days, spheres (> 75 μm diameter) were tallied and captured. Every 3 days, the culture medium was refreshed, and oncosphere numbers were counted using the Leica digital camera. The serum-free DMEM-F12 medium (Life Technologies, Grand Island, NY) involves the B-27 Supplement (Life Technologies, Grand Island, NY), 20 ng/ml basic fibroblast growth factor (bFGF, Gibco), and 20 ng/ml Epidermal Growth Factor (EGF, Gibco).

### Immunofluorescence method

All paraffin-sections of mouse lung tumors were acquired from a Leica Cryostat. The tumor sections were incubated with one of the following polyclonal antibodies: rabbit YAP1 antibody, rabbit SOX2 antibody, and rabbit CD133 antibody (all at 1:100 dilution, CST, Danvers, MA). After washing with the PBS for at least three times, the sections were incubated with the Alexa-488-conjugated or Alexa-594-conjugated secondary anti-body (1:200 dilution, Life Technologies) for 1 h at room temperature. Furthermore, the nuclei were stained with 4′,6-diamidino-2-phenylindole (DAPI) (1:500 dilution, Beyotime Institute of Biotechnology, China). All images were taken using a confocal laser-scanning microscope (Leica TCS SP5 II, Germany).

### Tumor Xenograft Model

Lung squamous cells transfected with miR-NC mimics or miR-214-3p mimics were triple-washed in the PBS and then suspended in matrigel. Cells were subcutaneously injected into the armpit of each mouse. Every 2 days, each tumor diameter was measured. Tumor volume was calculated by the equation: tumor volume (mm3) = 1/2 × length (mm) × width mm2. After nearly 4 weeks, tumors were all collected for subsequent analysis. All male BALB/C nude mice (SLAC, Shanghai) were fed at the specific pathogen free (SPF) Animal Center of Shanghai Chest Hospital. All experiments were performed according to the guidelines.

### Orthotropic lung tumor model

Cells were suspended in matrigel and orthotropically injected into the left lateral lungs of the mice, as described with minor modifications.

### Statistical analysis

GraphPad Prism software was employed for statistical analysis. All results were presented as mean ± Standard error of the mean (SEM) where indicated formats least three independent replicate experiments. Statistical differences were investigated using the Student’s t-test, χ2 test, and repeated measures analysis of variance. The Kaplan–Meier method was performed to assess overall survival (OS), and the log-rank test was performed to analyze the curves. Finally, the Cox model was utilized to identify independent prognostic factors. The statistical significance was set at P < 0.05.

## Results

### MiR-214-3p is downregulated in LSCC and is significantly associated with better clinical prognosis

**Fig. 1:**
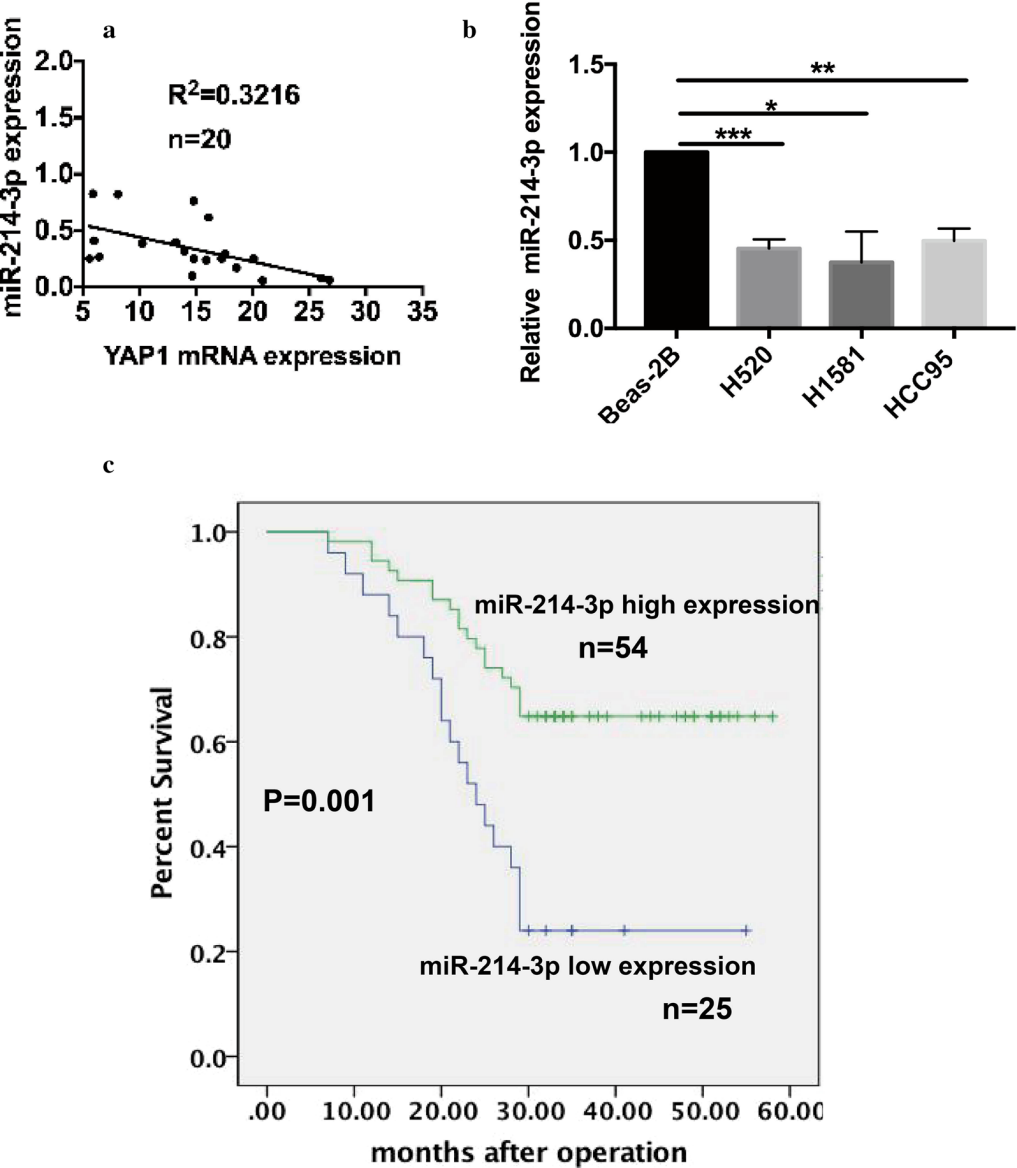
In lung squamous cell cancer (LSCC), the MiR-214-3p is significantly downregulated, which is related to better clinical outcomes. **a** Correlation analysis of miR-214-3p with YAP1 levels, as detected by qPCR. (n = 20). **b** Relative miR-214-3p levels in LSCC cell lines and the normal bronchial epithelial cell line BEAS-2B was used as the control. **c** Kaplan-Meier analysis of overall survival, relying on miR-214 levels in 79 LSCC patients, showed patients with higher miR-214 levels possessed a more favorable clinical outcome than patients with lower miR-214 levels (P = 0.001)

While YAP1 is elevated, the miR-214-3p is downregulated in tumor species compared to the matched normal bronchial epithelium using q-PCR analysis [19, 20]. In this context, our further analysis found a robust negative correlation between the miR-214-3p expression and YAP1 mRNA expression in LSCC species (n = 20, Fig. 1a). Next, we detected the miR-214-3p RNA levels in some LSCC cell lines and we observed the reduction of miR-214-3p in LSCC cell lines normalized to the normal bronchial epithelial cell line BEAS-2B accordingly (Fig. 1b). Then, we explored the relationship between miR-214-3p and corresponding clinical features. Patients were divided into two groups according to the median level of the miR-214-3p expression. We found the association of high miR-214-3p expression to smaller tumor size and lower Tumor Node Metastasis (TNM) stage (Table 1). There was no significant relationship between miR-214-3p and other clinical features, such as age and gender. Furthermore, Kaplan–Meier (KM) survival curves indicated that patients with high miR-214 expression possessed better clinical outcomes (P = 0.001, Fig. 1c, n = 79). The Cox regression analysis model revealed that the miR-214 expression and tumor size were independent prognostic indicators for LSCC (Table 2). In summary, these results suggested that miR-214-3p could have a critical role in LSCC initiation and progression.

**Table 1:** Correlations between miR-214-3p with clinicopathological features of LSCC patients

Variable	n	miR-214-3p		P
		Low (n = 25)	High (n = 54)	
Age (years)				
< 65	42	12	30	0.531
≥ 65	37	13	24	
Gender				
Female	41	16	25	0.143
Male	38	9	29	
Tumor size (cm)				
≤ 4.5	39	8	31	*0.036*
> 4.5	40	17	23	
Smoke				
Yes	31	6	25	0.059
No	48	19	29	
TNM stage				
I/II	59	15	44	*0.041*
III/IV	20	10	10	
Differential degree				
Poorly	52	20	32	0.071
Well, moderate	27	5	22	

Low/high by the sample median. Pearson X^2^ test *p < 0.05 was considered statistically significant

**Table 2:** Univariate and multivariate analysis of different prognostic variables and overall survival (OS) in 79 LSCC patients

Variables	Univariate analysis		Multivariate analysis model	
	HR (95% CI)	P	HR (95% CI)	P
Age (< 65 vs ≥ 65)	0.663 (0.350–1.257)	0.208		
Gender (Female vs male)	0.851 (0.450–1.608)	0.619		
Tumor size (≤ 4.5 vs > 4.5)	0.235 (0.111–0.498)	< *0.001*	0.383 (0.158–0.928)	*0.034*
Smoke (yes vs no)	1.245 (0.654–2.372)	0.504		
TNM stage (I/II vs III/IV)	0.235 (0.123–0.448)	< *0.001*	0.508 (0.234–1.104)	0.087
Differentiation degree (well/moderate vs poorly)	2.316 (1.223–4.387)	*0.010*	1.520 (0.712–3.246)	0.280
miR-214-3p expression (low vs high)	0.331 (0.175–0.628)	*0.001*	0.489 (0.249–0.960)	*0.038*

### Increased miR-214-3p inhibits LSCC cell proliferation and CSC properties in vitro

**Fig. 2:**
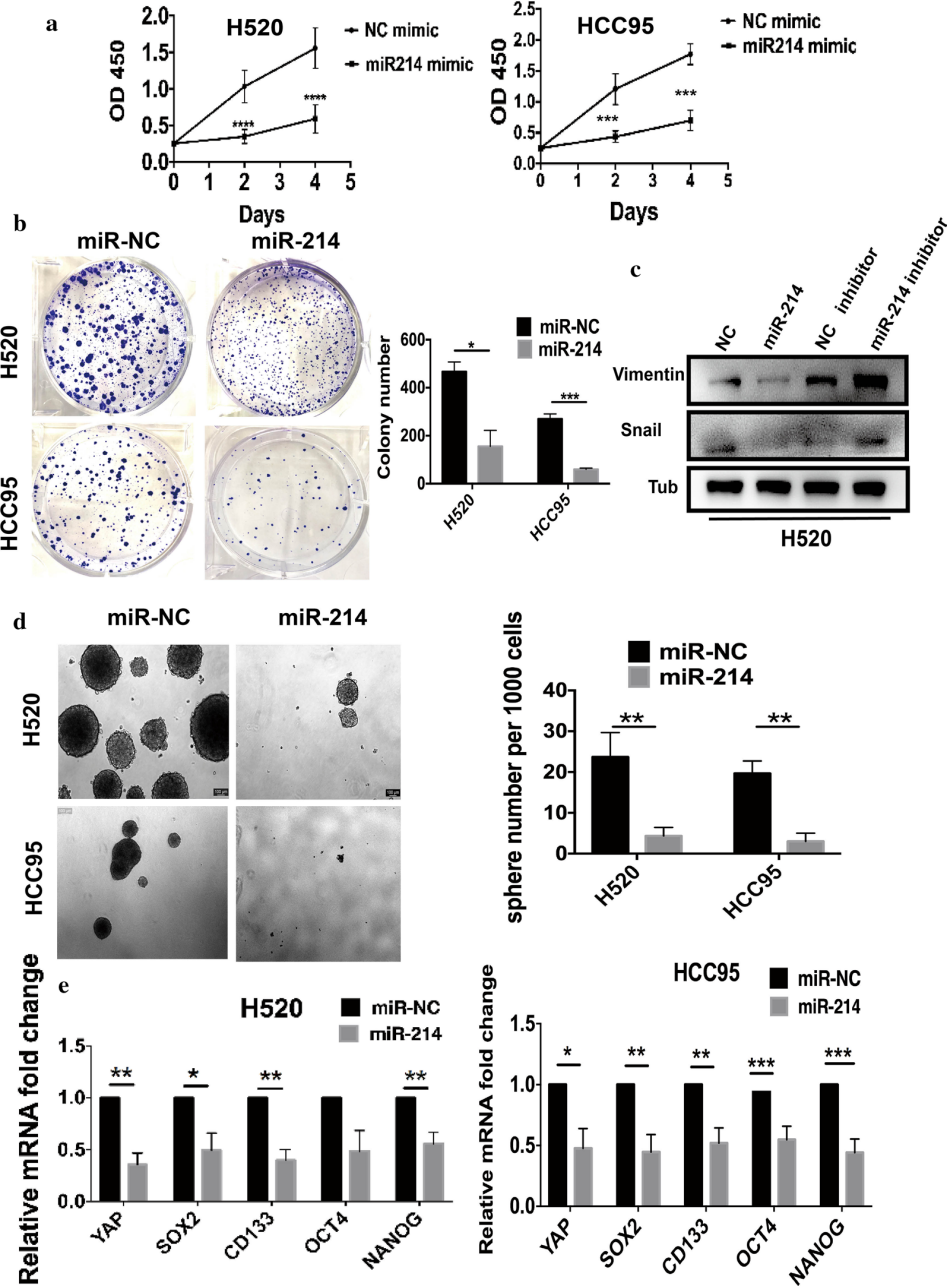
MiR-214-3p overexpression impairs LSCC proliferation and CSC properties in vitro. **a** CCK-8 assay indicated that MiR-214-3p reduced cell proliferation. **b** Colony-forming assays showed the effect of miR-214-3p. **c** Protein levels of Vimentin and Snail after overexpression or knockdown of miR-214-3p. **d** Sphere forming assays were used to examine the effects of miR-214-3p OE on LSCC. **e** The qPCR showed the expression of stem-related genes in both cells. Data are represented as mean and SEM from three independent experiments. * P < 0.05. ** P < 0.01. *** P < 0.001

To test the effects of miR-214-3p in lung cancer, we designed subsequent experiments. First, CCK-8 6assay demonstrated that the miR-214-3p overexpression (miR-214-3p OE) particularly inhibited proliferation in H520 and HCC95 cells compared to the NC groups (Fig. 2a). Vimentin and Snail are mesenchymal markers involved in many aspects of cellular organization, signaling, and proliferation. Western-bolt analysis proved that the miR-214-3p OE reduced the protein levels of Vimentin and Snail, while the miR-214-3p knockdown increased Vimentin and Snail protein levels accordingly (Fig. 2c). Additionally, the miR-214-3p OE remarkably inhibited colony and sphere formations compared with the control group (Fig. 2b and d). Moreover, q-PCR proved that the miR-214-3p OE substantially decreased the RNA expression of CSC-specific markers involving CD133, SOX2, OCT4, and NANOG (Fig. 2e). In addition, we detected the proteins level of CSC-specific markers, and the same conclusion was exhibited in Additional file 2: Figure S2.

### MiR-214-3p inhibits CSC properties of LSCC in vivo and decreases YAP1 protein level

**Fig. 3:**
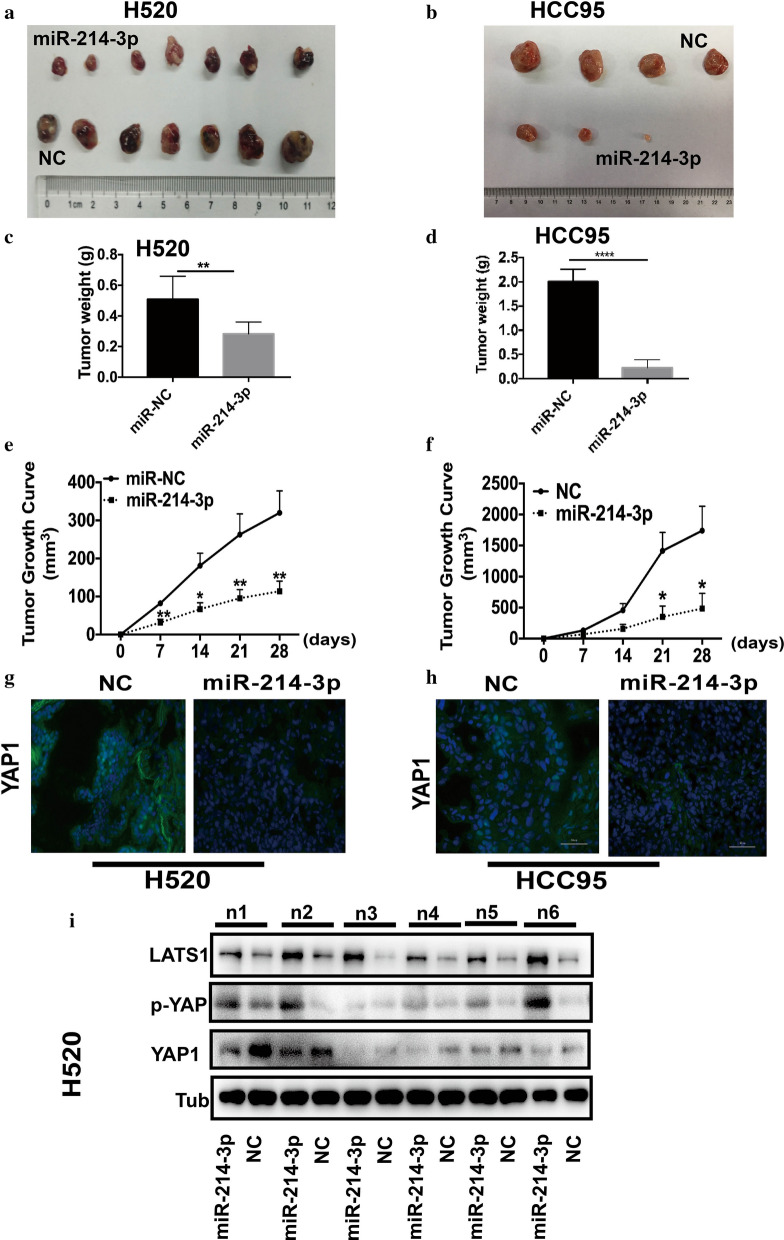
miR-214-3p impairs tumorigenicity of LSCC in vivo and activates the Hippo pathway decreases YAP1 protein level. **a** and **b** H520 and HCC95 cells transfected with miR-214-3p were injected into nude mice. **c** and **e** Tumor weight and tumor volume were analyzed in H520 cells. **d** and **f** Tumor weight and tumor volume were analyzed in HCC95 cells. **g** and **h** Fluorescence confocal microscope analysis demonstrated miR-214-3p overexpression decreased YAP1 levels of mice tumors. **i** Protein levels of Hippo components, after transfected with miR-214-3p in H520 cells. (At first, 7 mice models were established in each group, but 2 mice died in HCC95 groups).*p < 0.05, **p < 0.01, ***p < 0.001

Based on the above findings regarding in vitro, we illuminated the role of miR-214-3p in vivo. Then, H520 and HCC95 cells transfected with miR-214-3p or NC mimics were administered into flanks of nude mice. Figures 3a-f exhibited how tumor volume and weight in the miR-214-3p OE group were smaller than the NC group in both H520 and HCC95 mice models. Fluorescence confocal microscope analysis implied that miR-214-3p OE significantly decreased YAP1 levels in mouse models (Fig. 3g and h). The Hippo pathway regulates proliferation and apoptosis to control tissue size. Also, we observed that the miR-214-3p increased YAP1 phosphorylation and decreased YAP1 levels (Fig. 3i). Altogether, the results explained above demonstrated how miR-214-3p OE suppressed LSCC tumor progression by targeting YAP1.

**Fig. 4:**
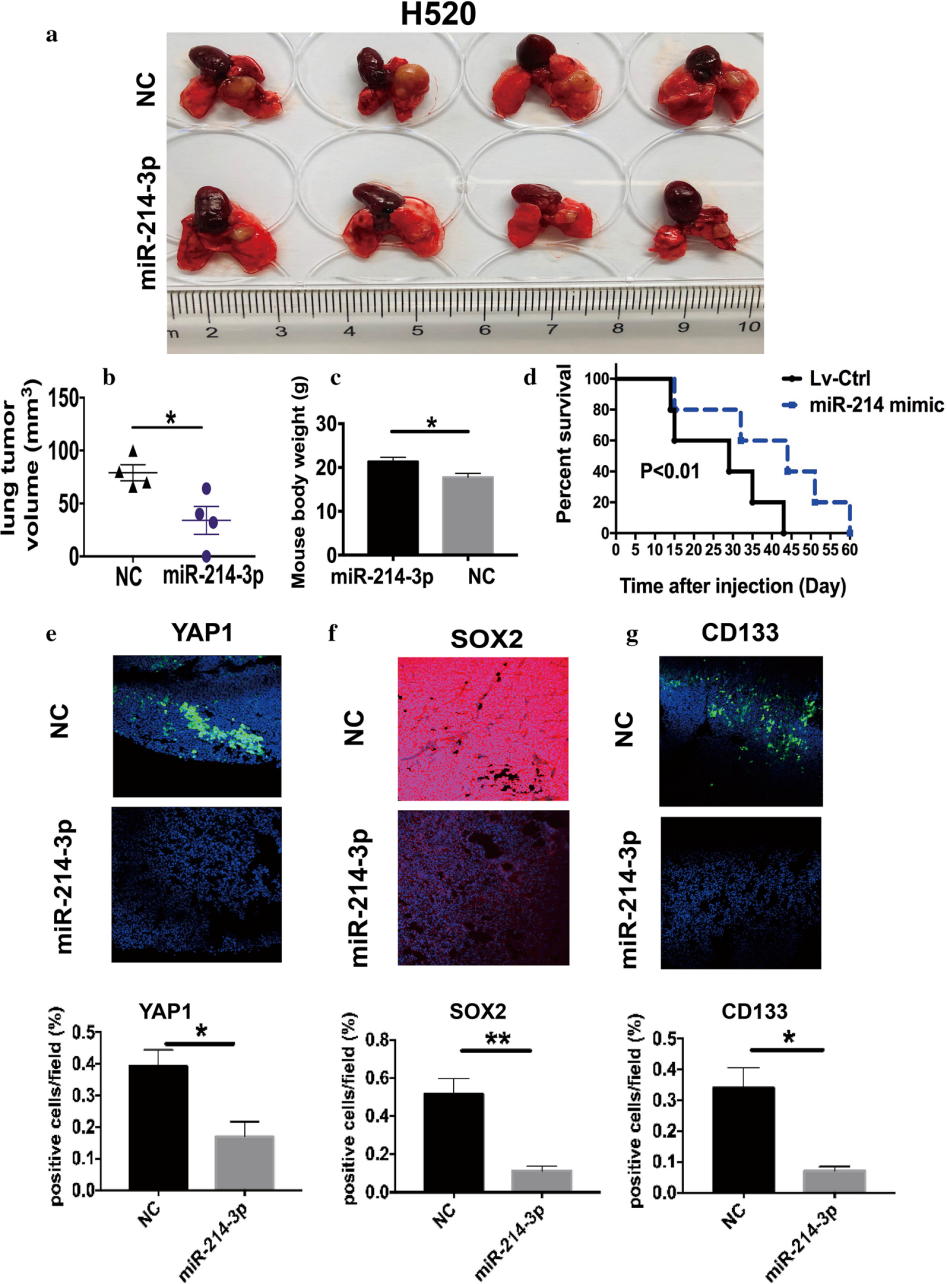
MiR-214 overexpression impairs LSCC tumor initiation and progression of orthotropic models. **a** Images of primary tumors in the left lungs at day 21 from each group after injection of H520 cells transfected with miR-214-3p mimics and NC mimics. **b** and **c** Mean primary tumor volume and body weight from two groups. (Error bars represent SEM; *p < 0.05). **d** Survival for the mice in each group evaluated. **p < 0.01. **e**–**g** Upper panel: Representative IF images of YAP1 and stem-related biomarkers, including SOX2 and CD133. Lower panel: Quantification of biomarker expression (Error bars represent SEM, N = 6; *p < 0.05; ** P < 0.01)

To further thoroughly verify the physiological function of miR-214-3p, we developed orthotropic mice models, which closely recapitulated human lung cancer. Cells were orthotropically administered into the left lateral lungs of nude mice. Compared to the NC groups, the miR-214-3p OE remarkably decreased tumor occurrence and tumor size (Fig. 4a and b). Figure 4c illustrated that mouse body weight in the miR-214-3p OE group is heavier than the NC group after injection, mainly caused by cachexia. Furthermore, the miR-214-3p OE significantly prolonged OS than the control group (Fig. 4d). In the future, larger animal models are needed for further research. Immunofluorescence (IF) staining assays showed a decrease of YAP1 and stem-related biomarkers, including SOX2 and CD133. This suggests the observable loss of proliferation and CSC properties in tumors (Fig. 4e–g).

### The miR-214-3p directly interacts with YAP1 in LSCC cells

**Fig. 5:**
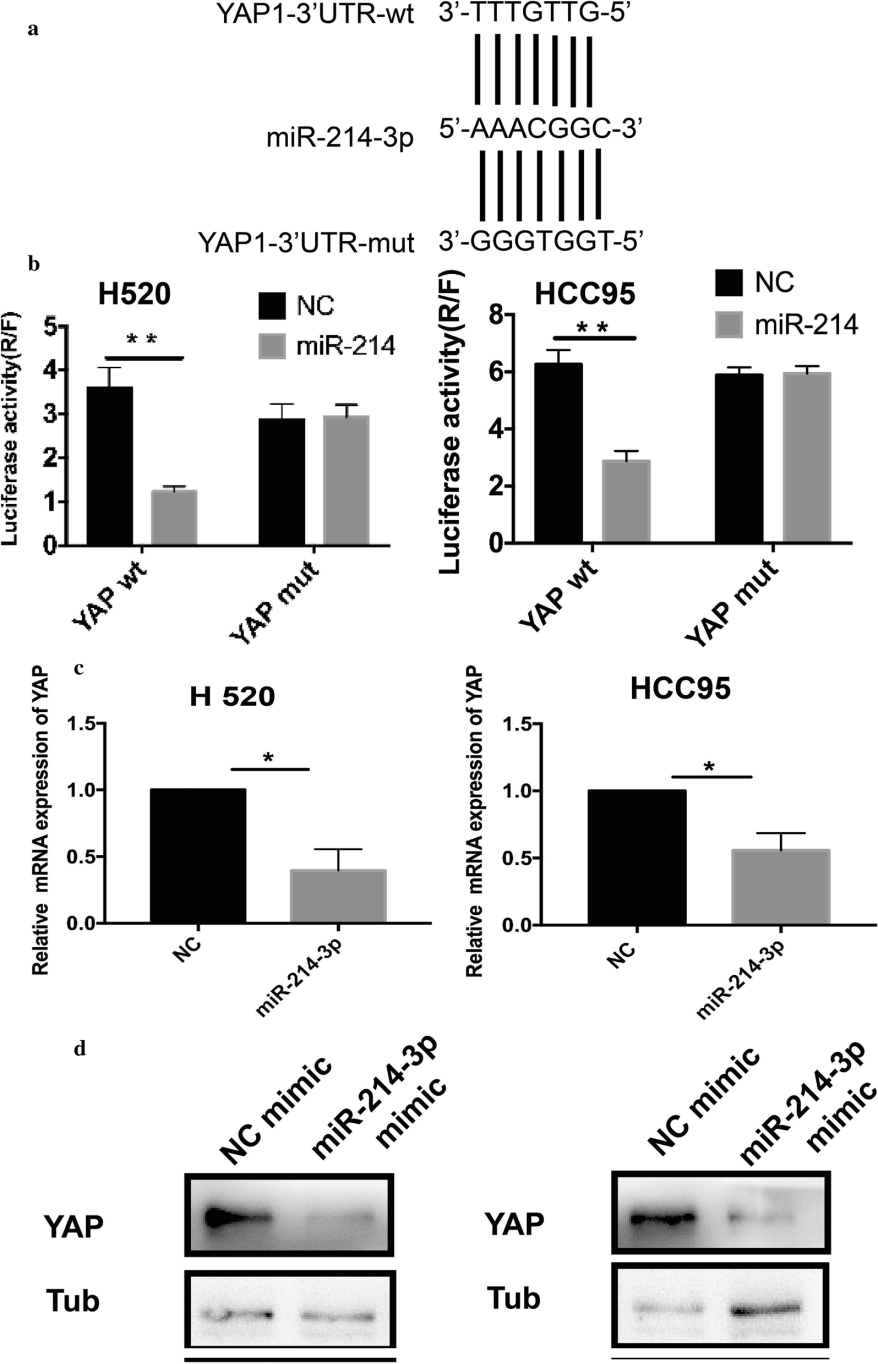
MiR-214-3p directly targets YAP1 in LSCC cells. **a** Binding sites of miR-214 in the YAP1 3′UTR. **b** Luciferase activity of each combination was assessed. **c** The YAP1 mRNA levels in H520 cell lines. **d** The YAP1 protein levels in LSCC cell lines transfected with miR-214 mimics. (*p < 0.05, **p < 0.01)

Given that the miR-214-3p is downregulated while YAP1 is up-regulated in LSCC patients, we speculated that YAP1 could be a target of miR-214-3p. Bioinformatic algorithms such as miRanda and TargetScan recommended that YAP1 possesses potential complementary sites for the miR-214-3p seed region in the 3′ Untranslated Region (3′UTR) (Fig. 5a). To verify this hypothesis, we transfected both the H520 and HCC95 cells with miR-214 or miR-214 inhibitors, and YAP1 mRNA and protein levels were measured. Results showed that the miR-214 OE significantly decreased YAP1 mRNA and protein levels in both cell lines, while miR-214 knockdown (KD) led to increased YAP1 expression (Fig. 5c and d). Furthermore, to determine the direct binding site between the miR-214-3p and YAP1, we performed a luciferase assay. Then, we constructed the wild type of and mutated seed sequences in the 3′ UTR of YAP1 mRNA. The YAP1-wt-3′UTR and its mutant type (YAP1-mt-3′UTR) were amplified and cloned downstream of a luciferase reporter gene in the pGL3-basic vector. The luciferase reporter experiment revealed that the co-transfection of pcDNAmiR-214 and YAP1-wt-3′UTR in H520 and HCC95 cells remarkably inhibited luciferase activity compared to the NC cells (Fig. 5b). However, there was no difference in the luciferase activity in cells that co-transfected with YAP1-mt-3′UTR and pcDNAmiR-214 (Fig. 5b). Therefore, we confirmed that miR-214-3p directly targets YAP1 by specifically binding on the 3′ UTR of YAP1 in LSCC cell lines.

### Rescue experiments illustrated that YAP1 liberated the growth and CSC properties inhibited by miR-214

**Fig. 6:**
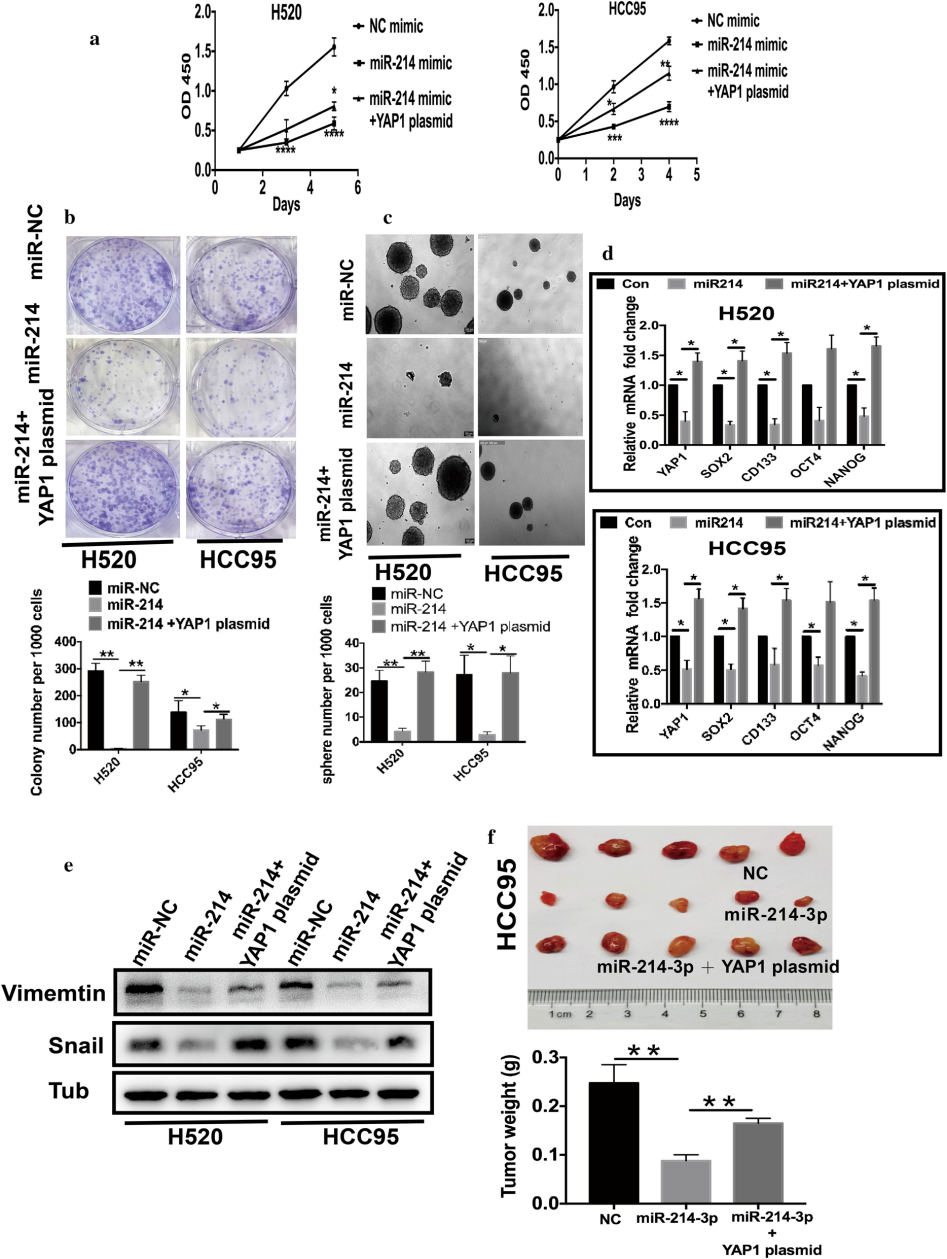
Rescue experiments confirm that YAP1 liberated the growth and CSC properties repressed by miR-214. miR-214-3p represses growth and CSC properties by inhibiting YAP1. **a** CCK experiments in LSCC cells transfected with different plasmids. **b** Colony formation in cells transduced with different plasmids. **c** Sphere-forming experiments in LSCC cells transfected with different plasmids. **d** Re-introduction of YAP1 rescued the reductions of CSC-specific markers in both cell lines. **d**, **e**. Vimentin and Snail protein levels were detected in LSCC cells transfected with different plasmids. **e, f**. In subcutaneous mice models, the re-expression of YAP1 remarkably decreased the anti-tumor effects of miR-214-3p

These findings encouraged us to explore whether the miR-214-3p repressed LSCC growth and CSC properties via YAP1. Our previous work revealed that YAP1 KD remarkably decreased sphere and colony formations and aldehyde dehydrogenase gene (ALDH) proportion. Preveiously, our group have proved that YAP1 KD substantially reduced stem-related genes levels of lung cancer cells [19]. These results suggested that YAP1 KD could mimic the role of miR-214-3p OE. Our findings above confirmed that silenced YAP1 led to the same phenotypes as miR-214-3p OE did, implying that miR-214-3p inhibited proliferation and CSC phenotype via YAP1. To validate this hypothesis, we performed a subsequent rescue experiment in H520 and HCC95 cells. Cells transfected with miR-214 were transduced with a pcDNA3.1-YAP1 plasmid. Interestingly, YAP1 OE partially rescued the proliferation capacity inhibited by the miR-214 (Fig. 6a). The YAP1 plasmid transduction liberated the inhibited growths of miR-214-3p OE colonies and spheres (Fig. 6b and c). We also found that re-introduction of YAP1 rescued the reductions of CSC-specific markers in both H520 and HCC95 cell lines (Fig. 6d). In addition, the re-introduction of YAP1 rescued the reductions of Vimentin and Snail protein expressions caused by the miR-214-3p OE (Fig. 6e). Furthermore, in mice models, the restoration of YAP1 expression significantly liberated the decreased proliferation and self-renewal abilities caused by the miR-214-3p OE in LSCC (Fig. 6f).

## Discussion

This study confirmed that YAP1 was overexpressed while miR-214-3p was downregulated in LSCC tissues, which was inversely correlated with each other. The KM survival curves indicated that patients with high miR-214 expression possessed better clinical outcomes than those with low expression. In this case, we initially reported that the miR-214-3p strongly modulated CSC phenotypes of LSCC in vitro and in vivo. We provided robust evidence on how miR-214-3p served as a tumor suppressor and a promising target in therapies against LSCC because it imitated and displayed excellent anti-tumor efficacy.

Like many other miRNAs, miR-214 has reportedly been abnormally expressed in various tumors. It plays a dual role by modulating tumor initiation based on the tumor types, either as oncogenes or tumor suppressors. As a tumor suppressor, miR-214 represses tumor progression in hepatocellular, gastric, ovarian, and cervical cancers. However, it is also reported to promote tumor migration in melanoma [21–25]. Furthermore, miR-214 acts as either an oncogene or a suppressor, depending on the genetic background or the cancer type. Interestingly, in lung cancer, Wu et al. reported that miR-214 OE significantly diminished the migration and invasion of non-small cell lung cancer (NSCLC) cells, while Long et al. found that miR-214 expression contributed to epithelial–mesenchymal transition (EMT) and metastasis of lung adenocarcinoma [26, 27]. However, the impact and regulatory mechanisms of miR-214 in LSCC remain to be extensively explored.

We demonstrated the role of miR-214 as a tumor suppressor and how miR-214 impaired the CSC properties of LSCC by gain- and loss-of-function assays. To our knowledge, the crucial role of miRNA is to regulate the expression of its downstream genes through the mRNA cleavage and/or by inhibition of translation, depending on the extent of complementarity with the 3′UTR of target genes. Our group previously confirmed that the miR-214-3p was downregulated, whereas YAP1 was upregulated in LSCC tissues [19]. In this context, we reported that miR-214 was inversely correlated with YAP1, which stimulated our attention and interest. Further investigations validated our hypothesis that YAP1 was a direct downstream gene of miR-214 in LSCC. First, miR-214 OE evidently reduced YAP1 mRNA and protein expression in LSCC. Second, ectopic miR-214 levels significantly impaired a luciferase reporter’s activity, which contains the 3′UTR sequence of YAP1. Third, rescue experiments showed that YAP1 re-expression abrogated the anti-tumor effect of miR-214 on LSCC to a certain extent.

To conclude, miR-214 acts as a critical regulator of CSC properties in LSCC. We also reported that miR-214 inhibited LSCC proliferation and CSC capacities via targeting YAP1 to activate the Hippo signaling pathway. The identification of tumor-specific miRNAs and target genes is necessary to understand the underlying mechanisms of LSCC initiation. This is of great value to design new strategies. In the future, miR-214-3p might become a promising prognostic marker. Finally, targeting the miR-214-YAP1 axis could be identified as a promising strategy in LSCC.

## Conclusion

Our data revealed that miR-214 could inhibit LSCC cell proliferation through the Hippo signaling pathway. Collectively, these findings provide new insight into the role of miR-214, which might serve as a novel therapeutic target for LSCC treatment.

## Supplementary information

**Additional file 1.** MicroRNA expression profile in LSCC human samples.

**Additional file 2.** MiR-214-3p OE substantially decreased the proteins level of CSC-specific markers.

## Data Availability

The datasets used and/or analyzed during the current study are available from the corresponding author on reasonable request.

## Abbreviations

miRNAs: microRNAs
LSCC: Lung squamous cell cancer
YAP1: Yes-associated protein1
CSCs: Cancer stem-like cells
LC: Lung cancer
AC: Lung adenocarcinoma
Mst1/2: Mammalian Sterile20-like Kinase1/2
Sav1: Salvador
FGFR1-amplified: Fibroblast Growth Factor Receptor1-amplified
NC: Negative Control
LATS1: Large tumor suppressor 1
p-YAP1: Phospho-YAP1
RIPA: Radio-Immunoprecipitation Assay
SDS-PAGE gels: Sodium dodecyl sulfate–Polyacrylamide Gel Electrophoresis gels
SYBR: Synergy Brands Synergy Brands
cDNA: complementary DNA
SOX2: SRY(sex determination region of Ychromosome)-related HMG-box 2
OCT4: Octamer-binding transcription factor-4
Opti-MEM: Reduced-Serum Medium
WT: Wide type
Mut: Mutation type
CCK8: Cell Counting Kit-8
OD value: Optical density value
DMEM: Dulbecco Modified Eagle Medium
PBS: Phosphate buffer saline
bFGF: Basic fibroblast growth factor
EGF: Epidermal Growth Factor
DAPI: 4,6-Diamino-2-phenyl indole
SPF: Specific pathogen free
SEM: Standard Error of Mean
OS: Overall survival
TNM: Tumor Node Metastasis
KM: Kaplan–Meier
OE: Overexpression
IF: Immunofluorescence
3′-UTR: 3′Untranslated Region
KD: Knowdown
ALDH: Aldehyde dehydrogenase gene
NSCLC: Non-small cell lung cancer
EMT: Epithelial–mesenchymal transition
