# Supplementary figures and images for: MicroRNA-214-3p inhibits the stem-like properties of lung squamous cell cancer by targeting YAP1
Source: Cancer Cell Int. 2020 Aug 27;20:413. doi: 10.1186/s12935-020-01506-2 (PMC7450582; doi:10.1186/s12935-020-01506-2)

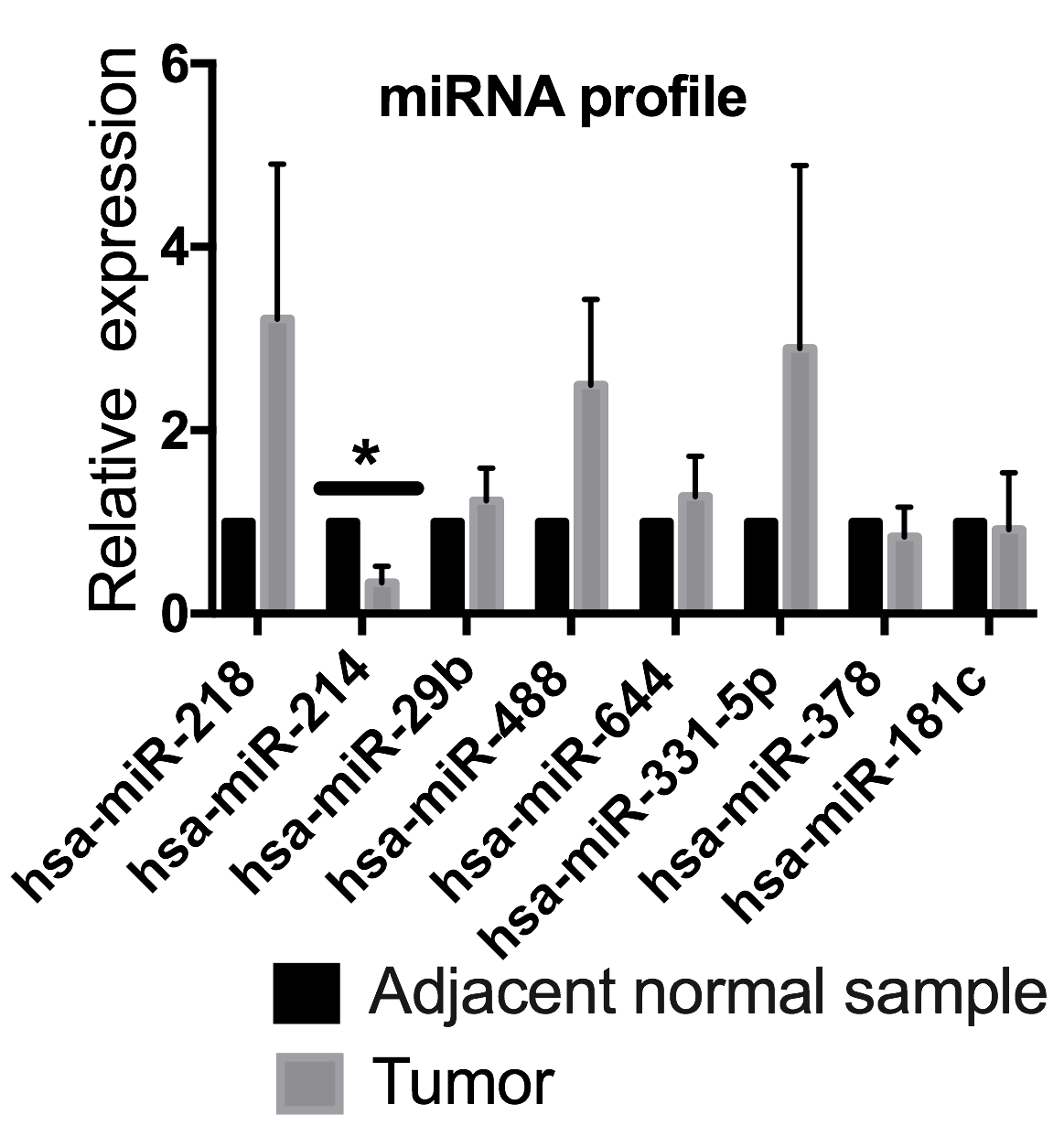

Supplement: Supplementary file 1 — Additional file 1. MicroRNA expression profile in LSCC human samples. [file 12935_2020_1506_MOESM1_ESM.tiff]

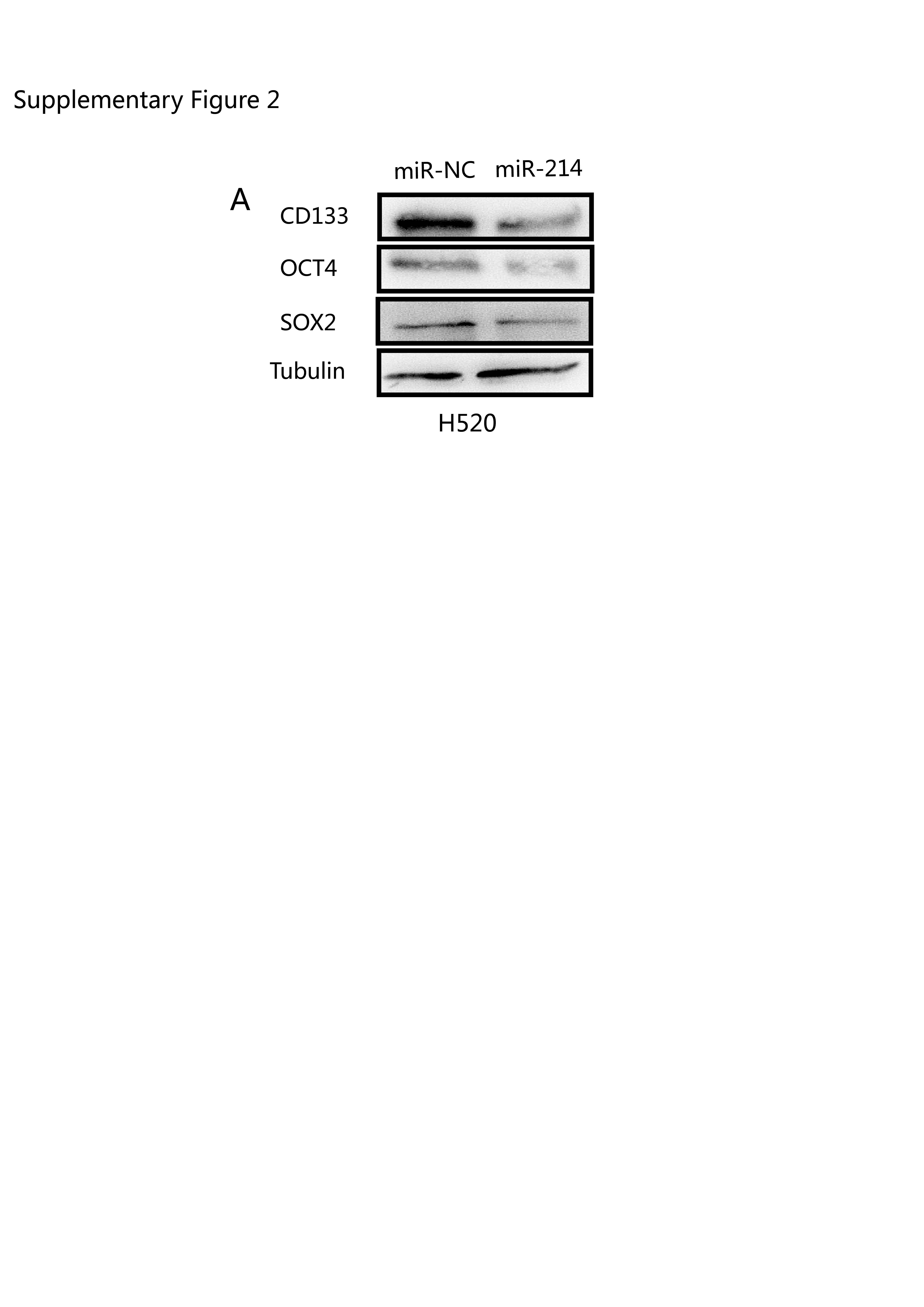

Supplement: Supplementary file 2 — Additional file 2. MiR-214-3p OE substantially decreased the proteins level of CSC-specific markers. [file 12935_2020_1506_MOESM2_ESM.jpg]
